# Characterization of macrophage activation after treatment with polysaccharides from ginseng according to heat processing

**DOI:** 10.1186/s13765-023-00774-6

**Published:** 2023-02-25

**Authors:** Sung Jin Kim, Seung-Hoon Baek, Ki Sung Kang, Myoung-Sook Shin

**Affiliations:** 1 College of Korean Medicine, Gachon University, Seongnam, 13120 Korea; 2 College of Pharmacy and Research Institute of Pharmaceutical Science and Technology (RIPST), Ajou University, Suwon, 16499 Korea

**Keywords:** Ginseng, Heat processing, Polysaccharides, Macrophage

## Abstract

The worldwide persistence of infectious diseases is a significant public health issue. Consequently, studying immunomodulatory ingredients present in natural products, such as ginseng, is important for developing new treatment options. Here, we extracted three different types of polysaccharides from white (P-WG), red (P-RG), and heat-processed (P-HPG) ginseng and analyzed their chemical properties and immunostimulatory activity against RAW 264.7 murine macrophages. Carbohydrates were the main components of all three polysaccharide types, while uronic acid and protein levels were relatively low. Chemical analysis indicated that the content of carbohydrates (total sugar) increased with processing temperature, while that of uronic acid decreased. Treatment with P-WG, P-RG or P-HPG stimulated nitric oxide (NO) production and increased tumor necrosis factor alpha (TNF-α) and interleukin (IL)-6 levels in RAW 264.7 macrophages, with P-WG showing the highest activity among the three polysaccharides. The expression of inducible NO synthase, which affects NO secretion, was highest in the macrophages treated with P-WG. Analysis of intracellular signaling pathways showed that mitogen-activated protein kinases (ERK, JNK, and p38) and NF-kB p65 were strongly phosphorylated by P-WG in macrophages but were only moderately phosphorylated by P-RG and P-HPG. Collectively, these results suggest that the polysaccharides isolated from ginseng undergo different changes in response to heat processing and display different chemical compositions and immune-enhancing activities.

## Introduction

Recently, infectious diseases, such as severe acute respiratory syndrome, Middle East respiratory syndrome, and coronavirus disease 2019, have occurred worldwide, and the number of patients has increased accordingly [1, 2]. The immune system plays a pivotal role in maintaining body homeostasis and preventing the diseases caused by infectious agents [3, 4]. Previous studies have reported the presence of several immunomodulatory compounds in natural products [5–7], including ginseng, which may provide new approaches to regulate the immune system and its response to infection.

Ginseng (*Panax Ginseng* C. A. Meyer) is a perennial plant belonging to the Araliaceae family that has been used as a traditional medicine in Asia for millennia. Ginseng is known to exhibit multiple pharmacological activities, including immune enhancement as well as anti-diabetic, anti-cancer, and anti-fatigue properties [8]. Ginseng contains ginsenosides, including Rb1, Rb2, Rc, Rd, Re, Rg1, and Rg3 [9, 10], and non-saponin fractions, such as amino acids, flavonoids, gintonins, polysaccharides, and organic acids [11, 12]. Ginsenosides are the main active compounds of ginseng and exhibit pharmacological activities such as the improvement of cardiovascular disease, protection of the nervous system and inhibition of cancer metastasis [13]. To improve the pharmacological activity of natural products, a heat-processing method was developed wherein the ingredients were altered depending on the heat-processing conditions used [14]. Ginseng is a representative natural product that is processed in this way to enhance its pharmacological activity. Ginseng is classified as white, red, or black according to the heat processing method used. Ginsenosides Rg3 and Rg5 have been reported to be newly generated after heat processing, whereas the contents of Re, Rf, and Rg1 have been shown to decrease and those of Rb1, Rb2, Rb3, Rc, and Rg increase [14].

However, not all pharmacological activities of ginseng are attributed to ginsenosides. Recently, natural product-derived polysaccharides have been reported to activate the systemic immune system through macrophage activation, which, in turn, can inhibit cancer metastasis or regulate the intestinal-mucosal immune system, including Peyer’s patches, following oral administration of plant-derived polysaccharides [5–7, 15]. The biological effects of natural polysaccharides have not been fully evaluated due to many technical challenges for purification and accurate structural analysis. Similar to the effect on structural changes on the glycosylic moieties in ginsenosides, heat treatment to ginseng may affect the monosaccharide composition and size of component polysaccharides. Structurally altered polysaccharides may also be the ingredients responsible for the enhanced biological activities or reduced side effects of heat-processed ginseng species.

The aim of this study is to evaluate the effect of heat treatment on the chemical properties and macrophage activation of crude polysaccharides extracted from ginseng. Crude polysaccharides were extracted from intact or heat-processed ginseng. The molecular weight and composition of the constituent monosaccharides were analyzed as the putative markers for the heat-induced structural changes in component polysaccharides. In addition, macrophage activation and intracellular signaling pathways of these polysaccharides were analyzed in RAW 264.7 macrophage cells.

## Materials and methods

### Cell lines

The RAW 264.7 murine macrophage cells were purchased from the Korea Cell Line Bank (Seoul, Korea), and maintained using DMEM containing 10% fetal bovine serum (FBS, heat-inactivated at 56 °C for 30 min) (ATCC, Manassas, VA, USA) and 1% penicillin/streptomycin (Gibco, Grand Island, NY, USA) at 37 °C and 5% CO_2_ condition.

### Antibodies

The antibodies used were those against stress-activated protein kinase/Jun amino-terminal kinase (SAPK-JNK), phospho-SAPK-JNK (Thr183/Tyr185), p38, phospho-p38 (Thr 180/Tyr 182) and p44/42 extracellular signal-related kinase (ERK), and phospho-p44/42 ERK. Antibodies against p65, phospho-p65 (Ser536), inducible nitric oxide synthase (DB65), and GAPDH were purchased from Cell Signaling Technology (Danvers, MA, USA). GAPDH antibody was used as loading controls*.*

### Plant material and preparation of P-WG, P-RG, and P-HPG

Dried root of four-year-old Panax ginseng (white ginseng [WG]) was purchased from Nonghyup (Seoul, Korea). A voucher specimen (A16077) was authenticated and deposited by Prof. Hong Pyo Kim, laboratory of pharmacognosy in College of Pharmacy in Ajou University. Red ginseng (RG) and heat-processed ginseng (HPG) were prepared by autoclaving WG (100 g) at 100 °C or 120 °C for 3 h, respectively, followed by oven drying at 60 °C. WG, RG, and HPG were extracted and fractionated according to our previously reported method, with minor modifications [16]. Briefly, dried WG, RG, and HPG (100 g each) were extracted three times with distilled water (500 mL) under reflux conditions for 3 h. Each aqueous extract was pooled and concentrated to a volume of 400 mL. After removing protein residues with Sevag reagent (CHCl_3_:BuOH = 4:1, v/v; 400 mL) via liquid–liquid extraction, the resulting aqueous layer was mixed with four volumes of ethanol and stored overnight at 4 °C. The precipitate was centrifuged (4500 × *g*, 5 min, 4 °C), reconstituted with distilled water, and then passed through a column filled with Di-anion HP-20 resin (4 × 30 cm). The eluate was dialyzed (CelluSep T2, MWCO 6000–8000; Membrane Filtration Products, Sequin, TX, USA) and lyophilized. Subsequently, polysaccharide extracts of WG (P-WG), RG (P-RG), and HPG (P-HPG) were used in all experiments.

### Molecular weight distribution

The molecular weight distribution of polysaccharide extracts was determined via high-performance gel filtration chromatography (HP-GFC) using a Flexar high-performance liquid chromatography system equipped with a reflective index detector (PerkinElmer, Waltham, MA, USA) [17–19]. The analytical column was a serial combination of a PL Aquagel-OH 30 column (300 × 7.5 mm, 8 μm particle size; PL1120-6830; Agilent Technologies, Santa Clara, CA, USA) and a PL Aquagel-OH 40 column (300 × 7.5 mm, 8 μm particle size; PL1149-6840; Agilent). The mobile phase was 0.2 M NaCl with a flow rate of 1 mL/min, and the column and detector temperatures were 25 °C and 40 °C, respectively. Pullulan standards (47.3–788 kDa; Showa Denko, Tokyo, Japan) were used as molecular weight markers. Solutions of the standards and polysaccharide extracts were prepared by dissolving them in distilled water at a concentration of 1 mg/mL. Each solution was filtered, and 30 μL was injected into the HP-GFC system. Data analysis including the determination of peak molecular weight (PMwt), weight average molecular weight (Mw), and number average molecular weight (Mn) was performed using TurboSEC software ver. 6.3.2 (PerkinElmer).

### Determination of total carbohydrate, uronic acid, and protein contents

The total carbohydrate, uronic acid and protein contents of the polysaccharide extracts were evaluated using the phenol–sulfuric acid method, m-hydroxydiphenyl assay, and Bradford assay, respectively, using glucose (phenol–sulfuric acid method), galacturonic acid (m-hydroxydiphenyl assay), and bovine serum albumin (Bradford assay) as standards [20].

### Monosaccharide composition analysis

Monosaccharide composition was determined via the 1-phenyl-3-methyl-5-pyrazolone (PMP) derivatization method using an ultra-performance liquid chromatography system (UHPLC; Shimadzu, Kyoto, Japan) equipped with a Shim-pack GIST column (2.1 × 100 mm, 2 μm particle size; Shimadzu). Derivatization and chromatographic analyses were performed following our previously reported method [16]. Briefly, 200 μg of polysaccharide extract was hydrolyzed with 100 μL of 2.5 M trifluoroacetic acid at 120 °C for 90 min in an autoclave. The hydrolysate was dried under a nitrogen stream and dissolved in 100 μL water. Approximately 400 μL of a 35% ammonia solution and 25 μL of a 0.5 M methanolic solution of PMP were then added, and the mixture was incubated at 70 °C for 30 min. The resulting product was dried under a nitrogen stream and dissolved in 1 mL water. The aqueous solution containing the PMP-derivatized monosaccharides was washed thrice with 1 mL chloroform and filtered through a 0.45 μm nylon filter, after which 1 µL of the filtered solution was injected into the UHPLC system. Mixtures of eight sugar standards (D-mannose, L-rhamnose, D-glucuronic acid, D-galacturonic acid, D-glucose, D-galactose, L-arabinose, and D-fucose) were prepared at concentrations of 0.01 to 0.25 nmol/tube, derivatized, and analyzed in the same manner. Mobile phase A was a mixture of an aqueous solution (0.045% KH_2_PO_4_, w/v; 0.05% triethylamine, v/v; final pH 7.5 with H_3_PO_4_) and acetonitrile (90:10, v/v), and mobile phase B was acetonitrile. Isocratic elution (A:B = 90:10) was performed at a flow rate of 0.3 mL/min. The column oven temperature was maintained at 35 °C. PMP-labeled monosaccharides were detected at a wavelength of 245 nm.

### Cell cytotoxicity assay

RAW 264.7 cells were seeded onto a 96-well microplate, at 1.0 × 10^5^ cells/well, and cultured at 37 °C and 5% CO_2_ for 24 h. The following day, P-WG, P-RG, and P-HPG were added at concentrations of 125, 250, 500, and 1000 μg/mL (diluted in DMEM) and incubated for 20 h. As a control, medium containing no sample was cultured in the same manner. The next day, 20 µL of EZ-Cytox (DoGENBio, Seoul, Korea) solution was added to the control and sample treatment groups and allowed to react for 30 min. Cell viability was measured using a microplate reader (Molecular Devices, San Jose, CA, USA) at a wavelength of 450 nm.

### Evaluation of NO production

RAW 264.7 cells were seeded onto a 96-well microplate and cultured at 37 °C in a 5% incubator overnight. The following day, P-WG, P-RG, and P-HPG were added at concentrations of 125, 250, 500, and 1000 μg/mL and incubated for 24 h. The culture supernatant was then harvested, and NO in the culture supernatant was measured. An equal amount of Griess reagent (Sigma-Aldrich, St. Louis, MO, USA) was added to the 96-well microplate and allowed to react in the dark for 10 min. The absorbance was measured at 550 nm using a microplate reader.

### Evaluation of TNF-α and IL-6 secretion in cells supernatants

RAW 264.7 cells were seeded onto a 96-well microplate, at 1.0 × 10^5^ cells/well, and cultured at 37 °C in a 5% incubator overnight. The following day, P-WG, P-RG, and P-HPG were added at concentrations of 125, 250, 500, and 1000 μg/mL and incubated for 24 h. The culture supernatant was harvested, and TNF-α and IL-6 secretion levels in the supernatants were determined using ELISA kits (eBioscience, San Diego, CA, USA) according to the manufacturer’s instructions.

### Evaluation of mRNA expression

RAW 264.7 cells were seeded onto a six-well plate (2.0 × 10^6^ cells/well) and cultured at 37 °C in a 5% incubator overnight. The following day, P-WG, P-RG, and P-HPG were added at concentrations of 125, 250, 500, and 1000 μg/mL and incubated for 12 h, after which the cells were washed three times with DPBS. Total RNA was extracted using the Qiagen RNeasy Mini Kit (QIAGEN, Hilden, Germany) according to the manufacturer's instructions. The extracted RNA was quantified using a NanoDrop spectrophotometer. cDNA was then synthesized using the RevertAid First Strand cDNA Synthesis Kit (Fermentas, Waltham, MA, USA), 1 μg of RNA, and Oligo dt primer in a final volume of 20 μL. The synthesized cDNA was diluted five times with DEPC water to a final volume of five times of the original. Quantitative reverse transcription-PCR was performed on a QuantStudio 3 real-time PCR system (Applied Biosystems). The primer sequences are listed in Table 1. The housekeeping gene *GAPDH* was used to normalize the expression of individual genes, and the changes in individual mRNA expression were analyzed using the _ΔΔ_CT method [21].

**Table 1:** Primer sequences used for quantitative reverse transcription-polymerase chain reaction (qRT-PCR)

Gene	Accession No	Primer sequences
Mouse *IL-6*	NM-031168	Sense 5´-GAGGATACCACTCCCAACAG-3´ Antisense 5´-AAGTGCATCATCGTTGTTCA-3´
Mouse *TNF-α*	NM_013693	Sense 5´-GCCTCTTCTCATTCCTGCTTG-3´ Antisense 5´-CTGATGAGAGGGAGGCCATT-3´
Mouse *GAPDH*	NM_008084	Sense 5´-GAGGATACCACTCCCAACAG-3´ Antisense 5´-AAGTGCATCATCGTTGTTGTTCA-3´

### Phosporylation of mitogen-activated protein kinase (MAPK), NF-κB and iNOS expression

RAW 264.7 cells were seeded onto a six-well plate (2.0 × 10^6^ cells/well) and cultured at 37 °C in a 5% CO_2_ incubator overnight. The following day, P-WG, P-RG, and P-HPG were diluted to 125, 250, 500, and 1000 μg/mL and used to treat the cells for either 30 min (for MAPK and NF-κB expression) or 18 h (for iNOS expression). The cells were then washed three times with cold DPBS, and cell lysates were harvested using radioimmunoprecipitation (RIP) assay (RIPA) buffer containing protease inhibitor cocktail, dithiothreitol (DTT), and phosphatase inhibitor cocktail. Finally, cell lysates were mixed with a 4 × Laemmli sample buffer (BioRad, USA) and heated at 95 °C for 5 min prior to sodium dodecyl-sulfate polyacrylamide gel electrophoresis analysis of the samples.

### Statistical analysis

All analysis was expressed as means ± SD of triplicate experiments. Statistical analysis was calculated from a one-way ANOVA followed by Tukey’s post-hoc test using Prism 8 (GraphPad Software, San Diego, CA, USA).

## Results

### Extraction and chemical properties of P-WG, P-RG and P-HPG

**Fig. 1:**
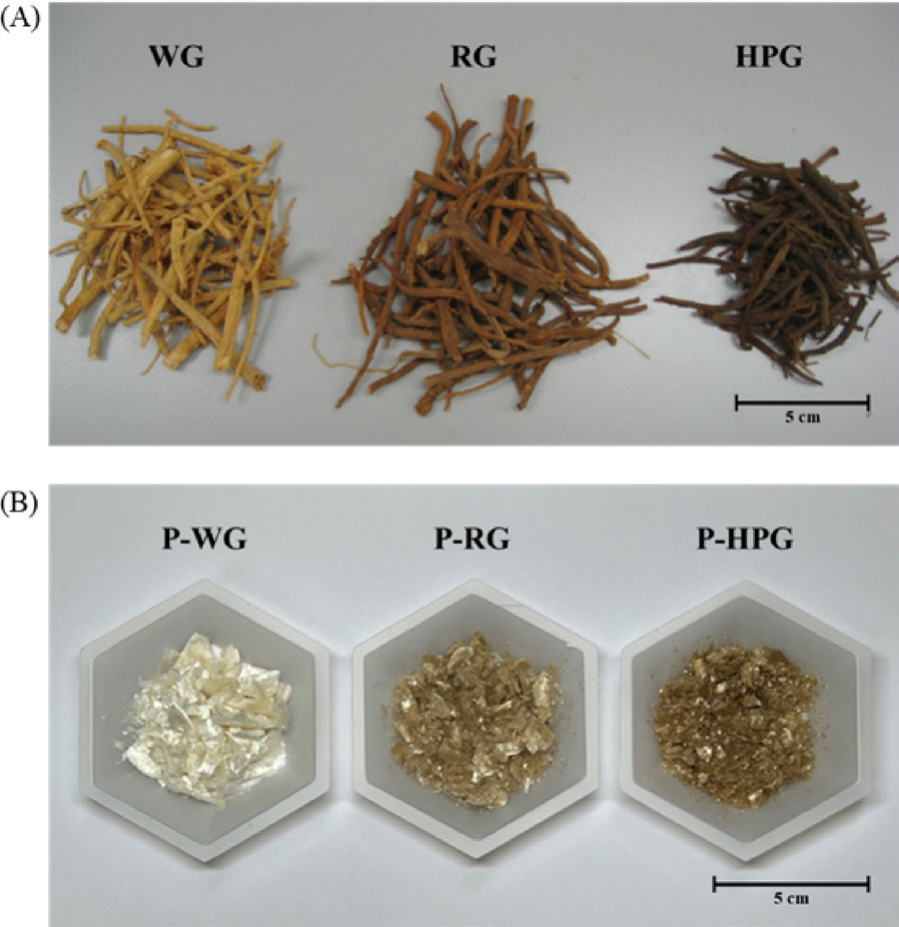
Image of original ginseng materials (**a**) and their polysaccharide extracts (**b**). *WG* white ginseng, *RG* red ginseng; *HPG* heat-processed ginseng, *P-WG* polysaccharide extract of WG, *P-RG* polysaccharide extract of RG, *P-HPG* polysaccharide extract of HPG

Images of the ginseng materials and their polysaccharide extracts are shown in Fig. 1. WG and P-WG were originally white but turned brown after treatment (Fig. 1: RG and HPG respectively). The degree of browning increased with the processing temperature. These browning effects may be due to products of the Maillard reaction formed in plant materials and foods exposed to high temperature [22].

**Fig. 2:**
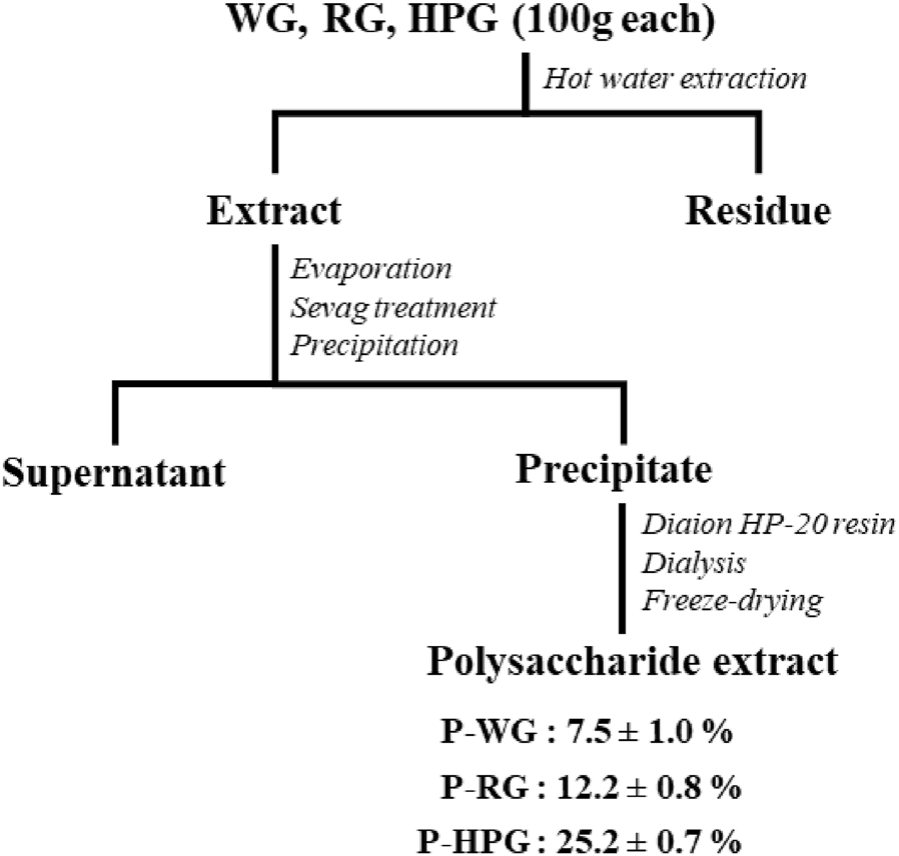
Preparation (extraction scheme) and chemical properties of P-WG, P-RG, and P-HPG

The overall procedure for the preparation of polysaccharide extracts from WG, RG, and HPG is shown in Fig. 2. The yields of P-WG, P-RG, and P-HPG were 7.5 ± 1.0%, 12.2 ± 0.8%, and 25.2 ± 0.7% (w/w), respectively. This suggests that the recovery of water-soluble polysaccharides increased with the processing temperature. Pretreatment with autoclaving has been reported to increase the extraction yield of structural polysaccharides, such as alginate, fucoidan, and laminarin, in the brown seaweed Laminaria digitata [23]. It also increases the extractability of cell wall polysaccharides in the green alga Caulerpa microphysa [24]. Autoclaving may lead to disruption of structural carbohydrates in the cell wall, thereby increasing their solubilization in water.

### Chemical properties and molecular weights of P-WG, P-RG and P-HPG

**Fig. 3:**
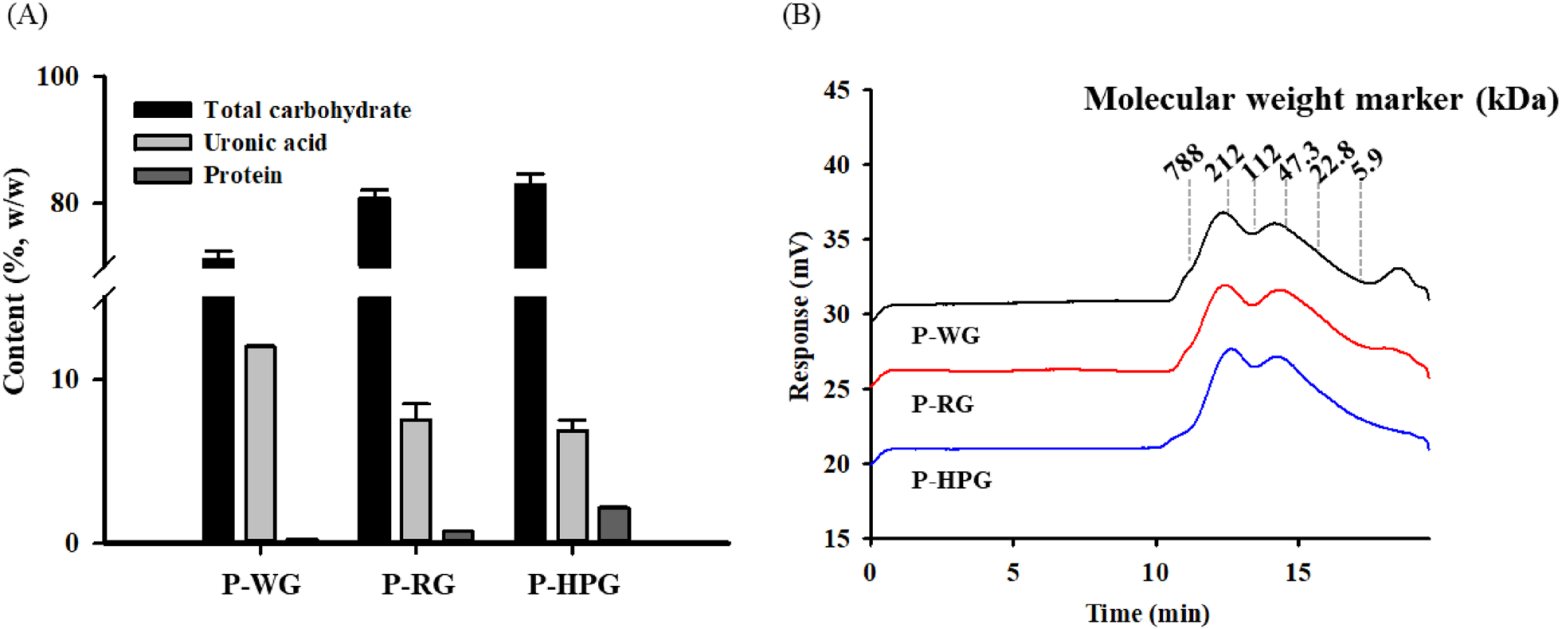
Preparation and chemical properties of P-WG, P-RG, and P-HPG. **a** Chemical properties of P-WG, P-RG, and P-HPG. Bars and error bars represent the mean and standard deviation, respectively. Break range on the y-axis is 15–70%. **b** HP-GFC chromatograms of P-WG, P-RG, and P-HPG. The elution times of pullulan molecular weight markers are indicated by dotted lines. The two major peaks of peak-1 and peak-2 are indicated. *HP-GFC* high-performance gel filtration chromatography

To determine the chemical composition of P-WG, P-RG and P-HPG, colorimetric analysis was used to determine the total carbohydrate, uronic acid, and protein levels (Fig. 3A). Carbohydrate was the major component in each extract (71.1 ± 1.3% for P-WG, 80.8 ± 1.4% for P-RG, and 82.9 ± 1.7% for P-HPG), while uronic acid and protein were relatively minor components. Carbohydrate and protein levels increased with increasing processing temperature, whereas uronic acid levels decreased. Molecular weight distribution was assessed using HP-GFC with pullulan molecular weight markers (Fig. 3B). All polysaccharide extracts showed a broad molecular weight distribution over the range of 5.9–788 kDa, which suggested that they mainly consisted of macromolecules. Then, the molecular weight associated with two major peaks (Fig. 3B, peaks 1 and 2) was determined. P-WG, P-RG, and P-HPG had molecular weights of 316.9 ± 8.5, 293.9 ± 3.9, and 246.2 ± 1.3 KDa, respectively, for peak 1 and molecular weights of 72.5 ± 1.8, 62.9 ± 0.5, and 66.6 ± 2.8 KDa for peak 2 (Table 2). These results suggest that autoclaving contribute*s* to lowering the size of polysaccharides.

**Table 2:** Molecular weight analysis of P-WG, P-RG, and P-HPG

Name	RT (min)	PMwt^1^ (KDa)	Mw^2^ (KDa)	Mn^3^ (KDa)
P-WG	12.3	322.9	392.4	302.1
	14.2	71.2	59	35.5
P-RG	12.4	296.6	367.1	285.7
	14.3	62.5	54.4	31.8
P-HPG	12.6	247.1	346.8	258.6
	14.2	68.5	49	22.6

^1^The peak molecular weight

^2^The average molecular weight

^3^The number average molecular weight

### Monosaccharide composition of P-WG, P-RG and P-HPG

**Fig. 4:**
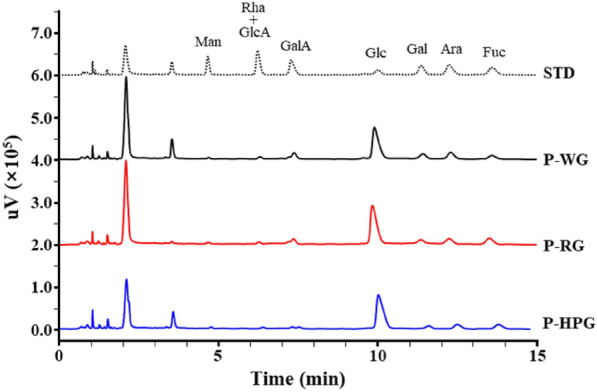
Preparation and chemical properties of P-WG, P-RG, and P-HPG. Overlaid chromatogram of monosaccharide composition analysis. The PMP-labeled standards (0.1 μmol mix/tube) and each sample (200 μg/tube) were analyzed using ultra- performance liquid chromatography (UHPLC); STD, Monosaccharide standard material; *Man* mannose, *Rha* rhamnose, *GlcA* glucuronic acid, *GalA* Galacturonic acid, *Glc* glucose, *Gal* galactose, *Ara* arabinose, *Fuc* fucose

The monosaccharide compositions of P-WG, P-RG, and P-HPG were determined using PMP derivatization and UHPLC analysis (Fig. 4). The monosaccharides were released by hydrolysis of the glycosidic bonds and derivatized using PMP. The PMP-labeled sugars were analyzed via UHPLC and UV detection.. Glucose was the dominant sugar in all extracts (Fig. 4), at 56.7% for P-WG, 57.6% for P-RG, and 65.7% for P-HPG. However, other sugars were present in lesser amounts. Glucose levels increased with the processing temperature, whereas the levels of the other sugars showed a decreasing trend. Taken together, these results suggest that autoclaving of ginseng lead*s* to chemical modifications of the extracts, which mainly consist of macromolecular polysaccharides. P-WG, P-RG, and P-HPG were used for subsequent biological assays.

### Effects of P-WG, P-RG and P-HPG on the viability of RAW 264.7 Cells

**Fig. 5:**
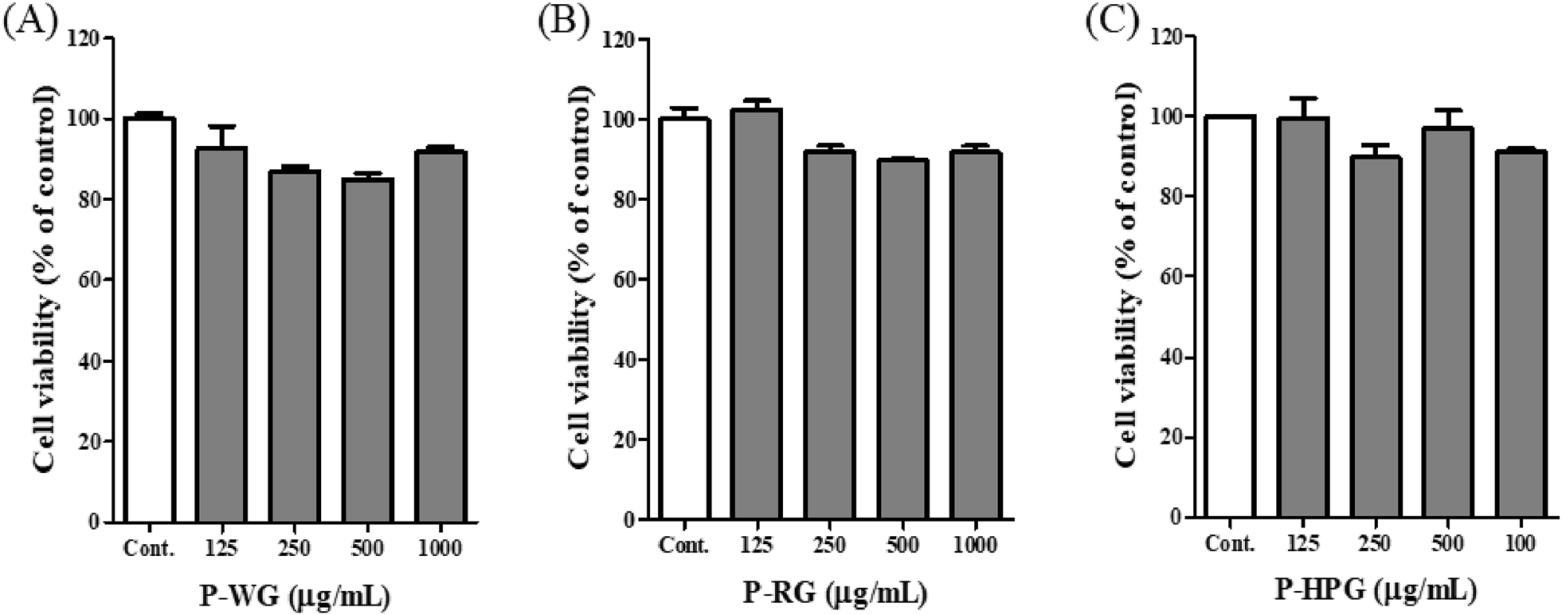
Effects of P-WG, P-RG, and P-HPG on the viability of RAW 264.7 cells. Cells were treated with either **a** P-WG, **b** P-RG, or **c** P-HPG at concentration*s* of 125, 250, 500 or 1000 μg/mL. Cell viability was assessed using an EZ-Cytox cell viability assay kit. All data are presented as mean ± standard deviation (SD), with n = 3

To evaluate the cytotoxicity of P-WG, P-RG, and P-HPG, a cell viability assay was performed using RAW 264.7 cells (Fig. 5A–C). When RAW 264.7 cells were treated with P-WG, P-RG or P-HPG at 125, 250, 500 or 1000 μg/mL, no significant difference in cell viability was observed relative to that in the control group. Therefore, the three types of ginseng polysaccharides were considered to be noncytotoxic at these concentrations. Subsequent experiments were conducted using 125, 250, 500, and 1000 μg/mL of P-WG, P-RG, and P-HPG.

### Stimulation of NO release by P-WG, P-RG and P-HPG in RAW 264.7 cells

RAW 264.7 cells were treated with P-WG, P-RG, and P-HPG for 22 h, and NO production evaluated. Lipopolysaccharide (LPS) was used as a positive control for monitoring macrophage activation [25–27]. After P-WG treatment at 125, 250, 500 or 1000 μg/mL, NO production increased at all concentrations. However, P-RG and P-HPG treatments only increased NO production significantly in the 500 μg/mL and 1000 μg/mL treatment groups. Therefore, as revealed by the treatment of macrophages with the three types of ginseng polysaccharides (P-WG, P-RG, and P-HPG) using different heat treatment processing methods, NO production is dependent on the processing conditions.

### Effects of P-WG, P-RG and P-HPG on iNOS expression in RAW 264.7 cells

**Fig. 6:**
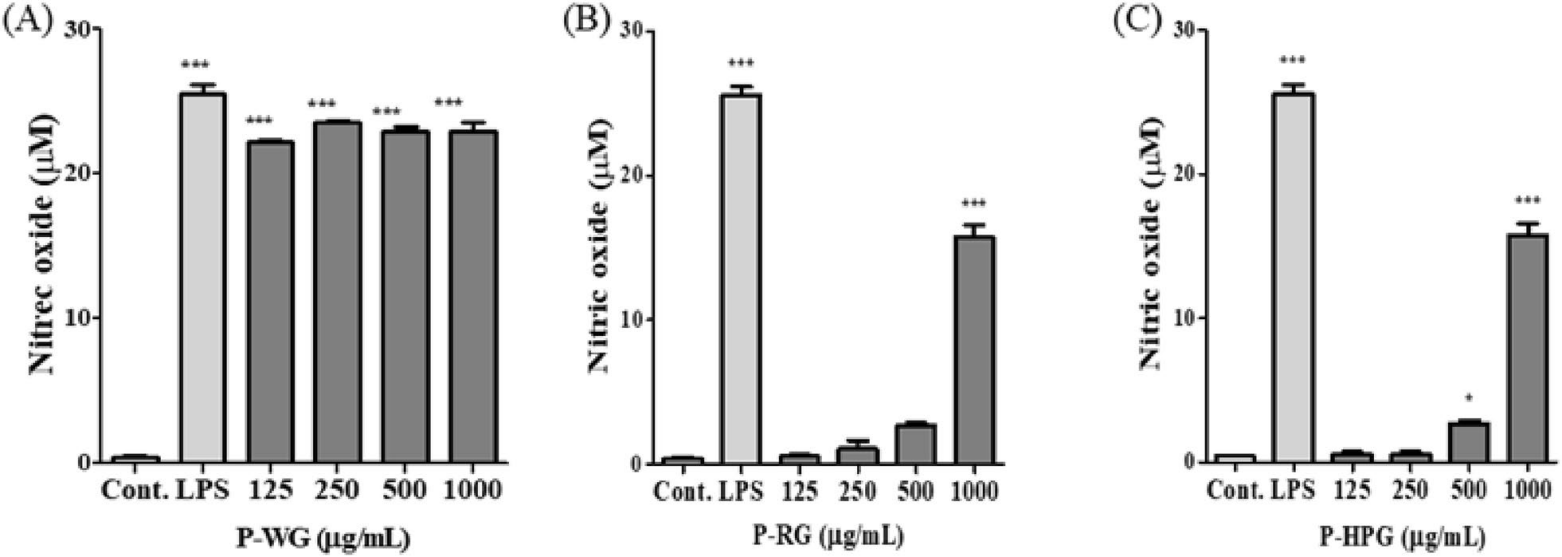
Effects of P-WG, P-RG, and P-HPG treatments on inducible nitric oxide synthase levels in RAW 264.7 macrophages. RAW 264.7 macrophages were stimulated with P-WG, P-RG, or P-HPG (125, 250, 500, or 1000 mg/mL) for 18 h, after which iNOS levels were evaluated. **a** iNOS levels determined via immunoblotting. **b** Quantification of iNOS/GAPDH intensity using the ImageJ software. All data are presented as mean ± SD, with n = 3, * P < 0.05 and *** *P* < 0.0001 compared to control

**Fig. 7:**
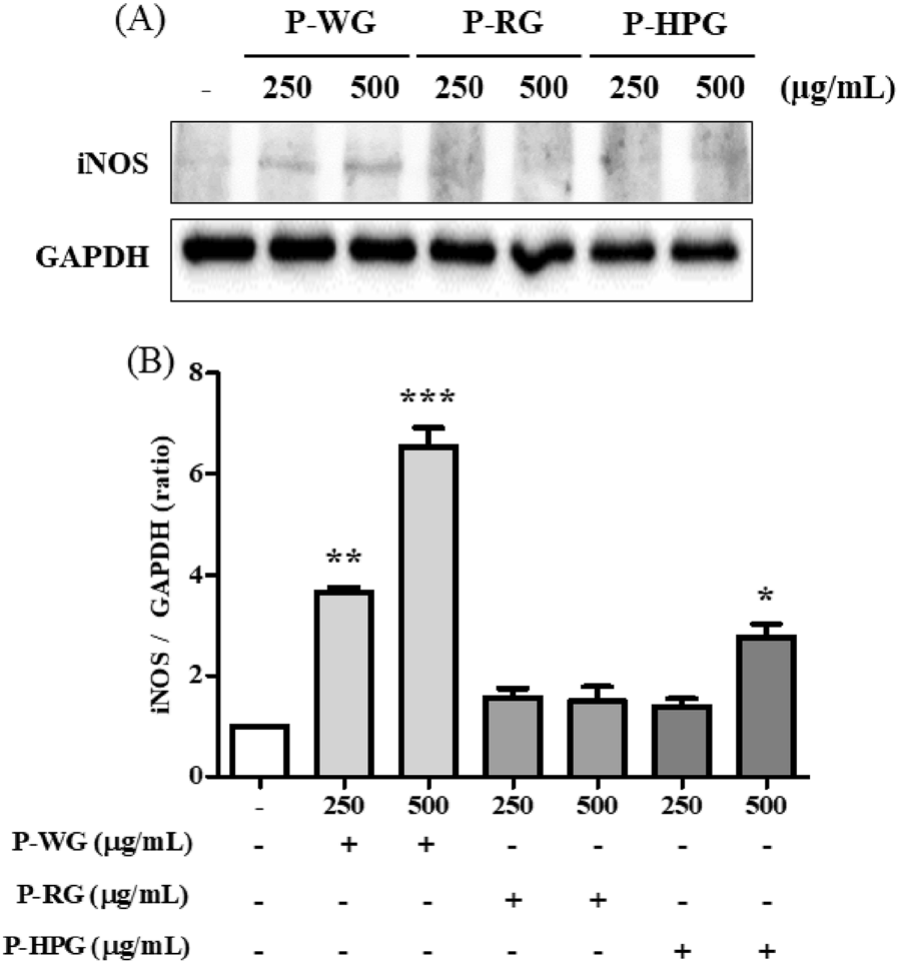
Effects of P-WG, P-RG, and P-HPG on nitric oxide (NO) production in RAW 264.7 macrophages. RAW 264.7 macrophages were stimulated with either **a** P-WG, **b** P-RG, or **c** P-HPG at concentrations of 125, 250, 500, or 1000 μg/mL for 22 h. Cell supernatants were harvested for NO production analysis (a–c). All data are presented as mean ± SD, with n = 3, *P < 0.05 and ***P < 0.0001 compared to control

NO is produced by iNOS. Hence, we investigated the protein levels of iNOS after P-WG, P-RG, and P-HPG treatment for 18 h in RAW 264.7 cells. iNOS protein levels increased significantly after treatment with 250 or 500 μg/mL of P-WG in a concentration-dependent manner (Fig. 6A, B). However, no change in iNOS levels was observed after P-RG or P-HPG treatment, similar to the effects on NO production shown in Fig. 7. Based on these results, it can be hypothesized that macrophages may be activated by treatment with P-WG, a white ginseng polysaccharide.

### Effects of P-WG, P-RG and P-HPG on TNF-α and IL-6 expression in RAW 264.7 cells

**Fig. 8:**
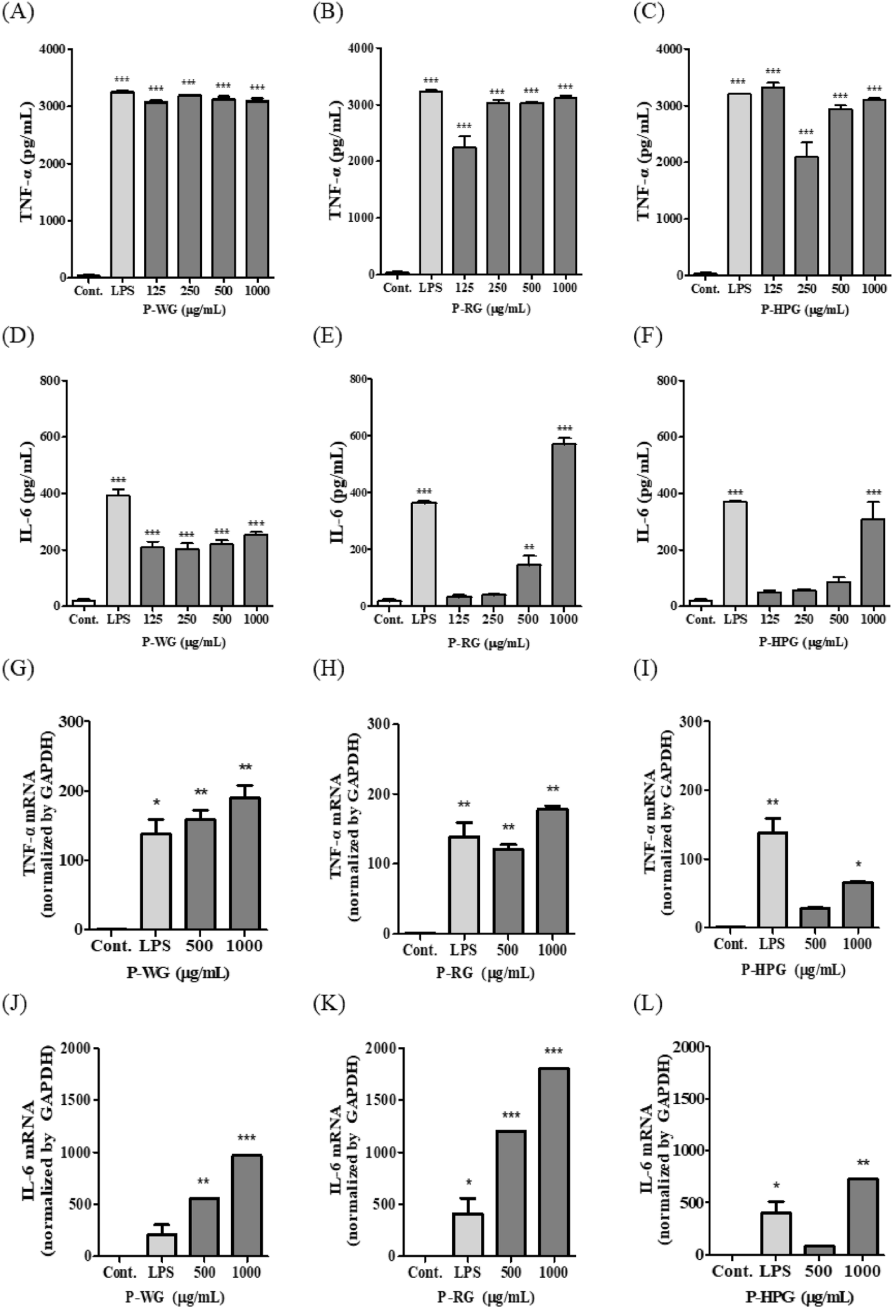
Effects of P-WG, P-RG and P-HPG on TNF-α and IL-6 expression in RAW 264.7 Cells. Cells were stimulated with P-WG (a and d), P-RG (b and e), or P-HPG (c and f) at 125, 250, 500, or 1000 μg/mL for 22 h. Cells supernatants were harvested, and TNF-α and IL-6 secretion was measured using commercial TNF-α and IL-6 ELISA kits, respectively. Cells were stimulated with P-WG (g and j), P-RG (h and k), or P-HPG (i and l) at 500 or 1000 μg/mL for 12 h. Cells were collected to determine TNF-α and IL-6 mRNA levels via quantitative reverse transcription-PCR. All data are presented as mean ± SD, with n = 3, ***P < 0.0001 and **P < 0.001 compared to control

Here, we investigated the effects of the three polysaccharide types (P-WG, P-RG and P-HPG), isolated from different heat-treated ginsengs on TNF-α and IL-6 cytokine production in RAW 264.7 cells. RAW 264.7 cells were treated with P-WG, P-RG, and P-HPG for 22 h, and TNF-α and IL-6 levels were measured using ELISA. TNF-α secretion was increased by LPS treatment (positive control) and by treatment with P-WG, P-RG, or P-HPG in all concentration groups (Fig. 8A–C). In contrast, IL-6 secretion was increased by P-WG at all concentrations but was only increased by P-RG and P-HPG at high concentrations (500 and 1000 μg/mL; Fig. 8D–F). To investigate the mRNA expression of TNF-α and IL-6, RAW 264.7 cells were treated with P-WG, P-RG, or P-HPG for 12 h. Both P-WG and P-RG significantly increased TNF-α and IL-6 mRNA levels at 500 and 1000 μg/mL in a concentration-dependent manner (Fig. 8G–I). In contrast, treatment with 1000 μg/mL P-HPG only slightly increased TNF-α and IL-6 mRNA levels (Fig. 8I, L).

### Effects of P-WG, P-RG and P-HPG on MAPK phosphorylation in RAW 264.7 cells

**Fig. 9:**
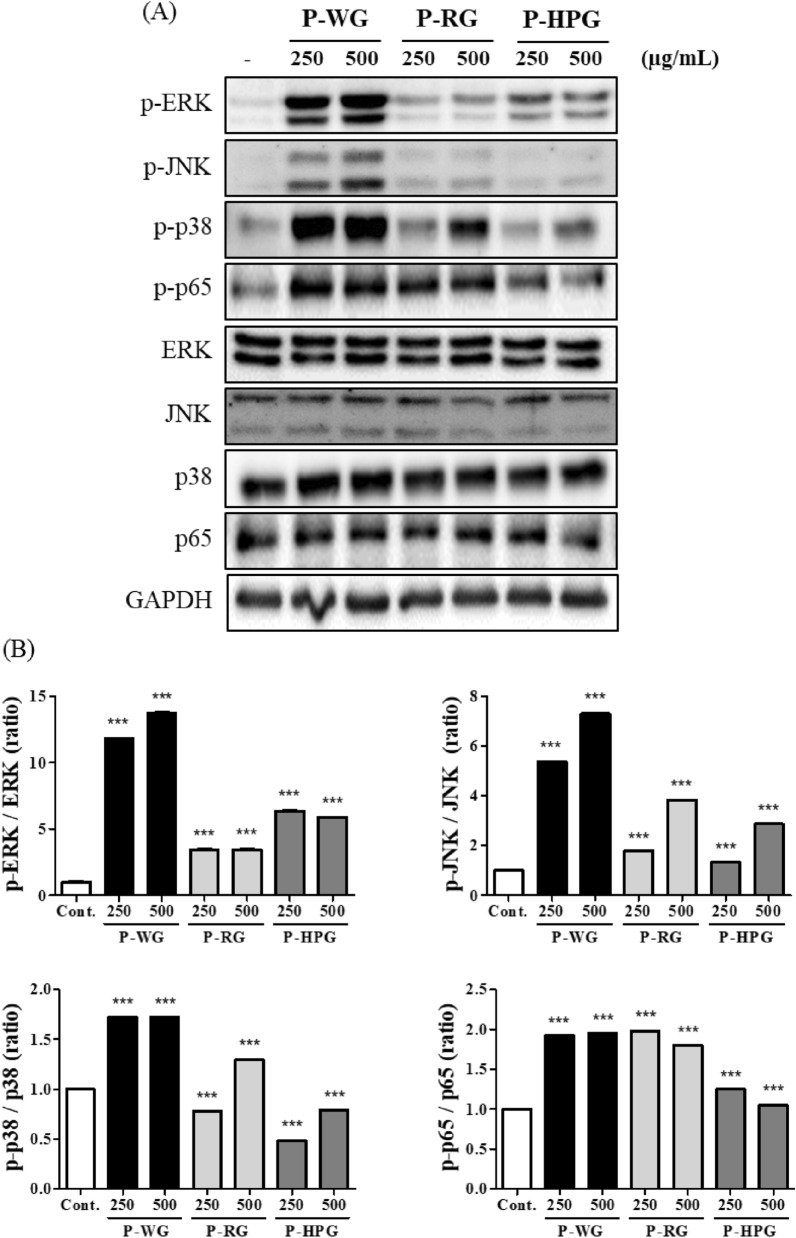
Effects of P-WG, P-RG, and P-HPG on the phosphorylation of MAPKs and NF-κB in RAW 264.7 cells. **a** RAW 264.7 macrophages were stimulated with P-WG, P-RG, and P-HPG at 250 or 500 μg/mL for 30 min. Phosphorylation and total protein levels of ERK, JNK, p38, and p65 in cell lysates from RAW 264.7 cells treated with P-WG, P-RG, and P-HPG were then determined. **b** Relative intensity of phosphorylated ERK, JNK, p38, and p65 after being normalized to ERK, JNK, p38 and p65, respectively, using the ImageJ software. Data are presented as mean ± SD, with n = 3. ***P < 0.0001 compared to the control group. *MAPK* mitogen-activated protein kinases, *NF-κB* nuclear factor kappa B

Next, RAW 264.7 cells were treated with P-WG, P-RG, and P-HPG for 30 min to evaluate their ability to increase the phosphorylation of MAPKs and NF-κB proteins. P-WG strongly increased the phosphorylation of ERK, JNK, and p38 proteins in a concentration-dependent manner, while P-RG and P-HPG induced small increase in the phosphorylation of ERK, JNK, and p38 (Fig. 8G, H). The total amounts of ERK, JNK, and p38 proteins were not changed by P-WG, P-RG, or P-HPG treatment. In addition, it was confirmed that phosphorylation of p65, an NF-kB subunit, was strongly induced by P-WG and P-RW treatment for 30 min but was only slightly induced by P-HPG treatment in RAW 264.7 cells. Phosphorylation due to polysaccharide (P-WG, P-RG, and P-HPG) treatment was quantified and graphed (Fig. 8H). These results are consistent with those shown in Figs. 6 and 8, indicating that P-WG may have the strongest macrophage-activating activity among the three polysaccharides (Fig. 9).

## Discussion

Heat treatment processing methods have been developed to enhance the pharmacological activity of natural products, so that the chemical composition changes depending on the heat treatment conditions [15, 16]. For example, *Rehmannia glutinosa*, a traditional Korean medicine, is divided into dried Rehmannia root and Sukhwanghwang (steamed nine times) according to heat treatment processing conditions, and the stachyose and catalpol contents are reduced. In Korea, ginseng is categorized into white, red, and black ginseng according to temperature processing [28, 29]. Analysis of the characteristics of ginseng polysaccharides according to temperature processing confirmed that the yields of polysaccharides, carbohydrates, and proteins increased with processing temperature (Fig. 2), while that of uronic acid decreased (Fig. 3a). In addition, the main component of the crude polysaccharide extracts of three ginsengs was confirmed to be glucose; glucose content also increased during heat treatment, whereas the levels of the remaining constituent sugars tended to decrease (Table 3). These results demonstrate that heat treatment of ginseng can induce chemical transformation of ingredients and affect the composition of extracts, which is mainly composed of high-molecular-weight polysaccharides. Further studies including the chromatographic purification, glycosidic linkage analysis, and spectroscopic assessment are needed to identify the precise structure of active polysaccharides as well as the heat-induced structural changes in ginseng polysaccharide.

**Table 3:** Chemical and monosaccharides composition of P-WG, P-RG, and P-HPG

	P-WG	P-RG	P-HPG
Chemical characteristics (%)			
Total carbohydrate	71.1 ± 1.3	80.8 ± 1.4	82.9 ± 1.7
Uronic acid	12.0 ± 0.1	7.6 ± 1.0	6.9 ± 0.7
Protein	0.21 ± 0.02	0.72 ± 0.02	2.17 ± 0.04
Monosaccharide composition (%)			
Man	Tr^1^	1.6 ± 0.2	1.6 ± 0.02
Rha + GlcA	Tr^1^	Tr^1^	Tr^1^
GalA	3.6 ± 0.9	3.0 ± 0.3	1.8 ± 0.1
Glc	57.4 ± 1.0	58.6 ± 1.4	66.5 ± 1.2
Gal	4.7 ± 0.1	3.7 ± 0.2	2.8 ± 0.1
Ara	5.0 ± 0.8	4.2 ± 0.4	3.4 ± 0.2
Fuc	3.6 ± 0.01	5.2 ± 0.6	4.4 ± 0.1

^1^Trace level

The immune system is a living system that maintains homeostasis by identifying and removing foreign substances invading the human body. It is categorized into innate and acquired immunity according to the specificity for foreign substances (antigens), with the former being an antigen-independent and fast-acting response. The innate immune system mainly involves physical defenses, such as skin or mucous membranes, and immune cells, such as natural killer cells and macrophages. Macrophages can remove foreign substances via phagocytosis or activate intracellular signaling pathways through pattern-recognition receptors present on the cell surface. Ultimately, they act as effector cells that present antigens to the adaptive immune system by inducing the secretion of various cytokines, such as IL-1β, IL-6, IL-12, and TNF-α [30, 31]. Nitric oxide produced by iNOS plays an important role in biological defense mechanisms, such as the removal of invading microorganisms, viruses, and pathogens [32, 33]. It was confirmed that NO production and iNOS both increased during treatment with P-WG, P-RG and P-HPG, with highest activity being observed during P-WG treatment. TNF-α is produced in lymphocytes and has been reported to induce tumor necrosis or improve resistance to infection by foreign antigens by acting alone or in combination with IL-1β in an in vivo immune response [34]. IL-6 and TNF-α cytokine secretion increased when P-WG, P-RG, and P-HPG were used, with the highest activity being observed after treatment with P-WG. In addition, the expression of TNF-α and IL-6 mRNA level were increased. In macrophages, specific receptors are expressed on the cell membrane, plant-derived polysaccharides can recognize these specific receptors. Therefore, receptors activate MYD88, TRAF-6, and IRAK1/4 and trigger a series of signaling pathways, such as MAPKs and NF-κB in macrophage cells [5, 18, 35]. Phosphorylated MAPKs migrate into the nucleus and act regulate transcription factors. They induce cytokine and NO production by activating AP-1 transcriptional regulatory proteins [36–38]. Meanwhile, NF-κB is a transcription factor that is inhibited by the non-phosphorylated form of IκBα in the cytoplasm. In activated macrophages, IκB kinase phosphorylates, leading to the degradation of IκBα, and NF-κB migrates to the nucleus. NF-κB then initiates gene transcription and induces the production of various cytokines [37–40]. When RAW 264.7 cells were treated with the polysaccharides derived from heat-processed ginseng, the phosphorylation of JNK, ERK, p38, and p65 proteins was highest during P-WG treatment.

Polysaccharides isolated from botanical sources have been shown to exert beneficial effects through modulating macrophage immune functions. However, the results of studies evaluating macrophage activity using only cell lines are controversial in terms of whether the polysaccharide has an immune-enhancing function or an inflammatory effect. Therefore, to explore the possibility of developing P-WG as an immunomodulator, further studies on immunomodulation, including animal experiments, are warranted.
